# Pretreatment Amide Proton Transfer‐Weighted Imaging Histogram Analysis Combined With ER‐Negative and HER2‐Positive Expression Predicts Pathologic Complete Response After Neoadjuvant Chemotherapy in Breast Cancer

**DOI:** 10.1002/cam4.71420

**Published:** 2025-12-07

**Authors:** Mingzhe Xu, Dongqiu Shan, Xuejun Chen, Renzhi Zhang, Chunmiao Xu, Jing Li, Zhiwei Shen, Yue Wu, Jinrong Qu

**Affiliations:** ^1^ The Department of Radiology The Affiliated Cancer Hospital of Zhengzhou University & Henan Cancer Hospital Zhengzhou China; ^2^ The Department of Radiology, National Cancer Center/National Clinical Research Center for Cancer/Cancer Hospital, Chinese Academy of Medical Sciences and Peking Union Medical College Beijing China; ^3^ Philips Healthcare Beijing China

**Keywords:** amide proton transfer, breast cancer, diffusion weighted imaging, neoadjuvant chemotherapy, pathological complete response

## Abstract

**Purpose:**

To evaluate the predictive value of pre‐treatment histogram analysis using APTWI, diffusion‐weighted imaging (DWI), and early contrast‐enhanced silhouette imaging in determining pathological complete response (pCR) post‐NAC in breast cancer, and to investigate whether combining immunohistochemical indicators enhances predictive accuracy.

**Materials and Methods:**

A retrospective continuous collection of 108 females with breast cancer who underwent NAC and pre‐treatment APTWI, DWI, and dynamic contrast‐enhanced imaging at our hospital. Clinical, MRI imaging, and pathological characteristics were analyzed for patients. NAC response was divided into pCR and non‐pCR. Tumor segmentation and histogram feature extraction were performed on APT, ADC, and early contrast‐enhanced silhouette images, and combined them with clinical features to construct an NAC efficacy prediction model. Diagnostic performance was assessed using receiver operating characteristic curves, with DeLong's test employed to compare areas under the curve (AUC).

**Results:**

In pCR group, mean, root‐mean‐square deviation, and 5th, 10th, 15th, 25th, 50th, 75th, 85th percentile of MTRasym, along with 1st percentiles of ADC were significantly higher in the pCR group than in the non‐pCR group (*p* < 0.05). Conversely, the interquartile range of MTRasym and 10th percentiles of ADC were significantly lower in the pCR group (*p* < 0.05). ER‐negative, HER2‐positive expression, and 5th percentile MTRasym value were identified as independent predictors of pCR post‐NAC (odds ratios, 0.16, 7.25, and 1.35, respectively). The combined diagnostic model demonstrated an AUC of 0.844, significantly outperforming individual parameters (*p* < 0.05).

**Conclusion:**

Pre‐treatment histogram analysis of MTRasym values derived from APTWI provides significant predictive value for pCR post‐NAC in breast cancer. The combined diagnostic model incorporating APTWI with ER and HER2 expression status further enhances diagnostic performance.

## 1 Introduction

Breast cancer is the most common cancer globally and remains the leading cause of cancer‐related mortality among women [1]. It exhibits high heterogeneity [2], characterized by distinct molecular subtypes, pathological grades, molecular markers, gene mutations, and immunological markers [3]. Comprehensive evaluation based on subtype and stage is essential for guiding treatment, which may include surgery, radiotherapy, chemotherapy, endocrine therapy, targeted therapy, and immunotherapy. Neoadjuvant chemotherapy (NAC) is a standard approach for locally advanced breast cancer, aimed at tumor downstaging to improve surgical resectability and enable breast conservation [4]. However, NAC response varies significantly, with fewer than 30% of patients achieving pathologic complete response (pCR) and approximately 5% experiencing disease progression during treatment [5]. Early and accurate assessment of NAC response is crucial to avoid ineffective chemotherapy and its associated toxicities, thus facilitating better treatment planning and surgical decisions.

Magnetic resonance imaging (MRI), recommended by the National Comprehensive Cancer Network (NCCN) breast cancer guidelines, offers high sensitivity and specificity in assessing NAC response [6]. Chemical exchange saturation transfer (CEST) imaging, an emerging non‐invasive molecular MRI technique, includes amide proton transfer‐weighted imaging (APTWI). APTWI enables noninvasive, radiation‐free detection of the exchange rate between endogenous free amide protons (‐NH) and hydrogen protons in water at 3.5 ppm, providing insights into cellular metabolic changes and pathophysiological processes [7, 8]. The signal intensity of APTWI is quantified using the asymmetric magnetization transfer rate (MTRasym) [9], which is positively correlated with protein concentration and pH levels in the surrounding environment [10]. While APTWI is widely used in brain tumor imaging [9, 11, 12], it remains a relatively new area of exploration in breast cancer. Previous studies have shown that APTWI can differentiate between benign and malignant breast tumors, with malignant lesions exhibiting significantly higher APTWI signals. Some research has also explored correlations between APTWI, molecular subtypes, and tumor immunohistochemistry [5, 13, 14]. A few studies have demonstrated its potential for predicting NAC response before treatment; however, these findings remain controversial [15, 16]. Additionally, most studies have involved small sample sizes (fewer than 72 cases), highlighting the need for validation through larger‐scale research. Diffusion‐weighted imaging (DWI) is a non‐invasive sequence that detects the free diffusion of water molecules within living tissues and has been widely used in tumor imaging. However, previous studies have shown inconsistent results regarding the value of its quantitative parameter, ADC, in predicting NAC response [17]. A previous study has also shown that early dynamic contrast‐enhanced MRI (DCE‐MRI) parameters such as enhancement rate and net enhancement correlated with NAC efficacy [18].

Histogram analysis provides a quantitative approach to assessing tumor heterogeneity by evaluating pixel grayscale frequency distribution. Recent advances in artificial intelligence and radiomics have introduced advanced image preprocessing and context‐aware spatial decomposition techniques, improving tumor segmentation and feature extraction in breast cancer imaging [19]. Additionally, synergistic feature selection and distributed classification frameworks have been developed to manage high‐dimensional medical data, enhancing diagnostic and predictive performance [20]. These developments highlight the growing role of computational analysis combined with quantitative imaging biomarkers in predicting treatment response. However, no previous studies have investigated post‐NAC pCR prediction in breast cancer using histogram analysis of APTWI or early post‐contrast silhouette images. In addition, previous studies have shown that immunohistochemical indicators, such as ER, PR, HER2 status, and Ki‐67 levels, are related to pCR in breast cancer [21].

### 1.1 Motivation and Objectives

This study aimed to investigate the predictive value of histogram analysis based on pre‐treatment APTWI, DWI, and early post‐contrast silhouette images in assessing the pathological response of breast cancer to NAC. Furthermore, it examines whether combining these methods with immunohistochemical indicators can improve predictive accuracy, thereby providing reliable diagnostic tools for guiding clinical decisions, optimizing treatment plans, and monitoring therapeutic efficacy.

## 2 Materials and Methods

### 2.1 Patients

This study was approved by the Institutional Review Committee of our hospital (No. NCT05063136). As a retrospective study, written informed consent from patients was not required.

**FIGURE 1:**
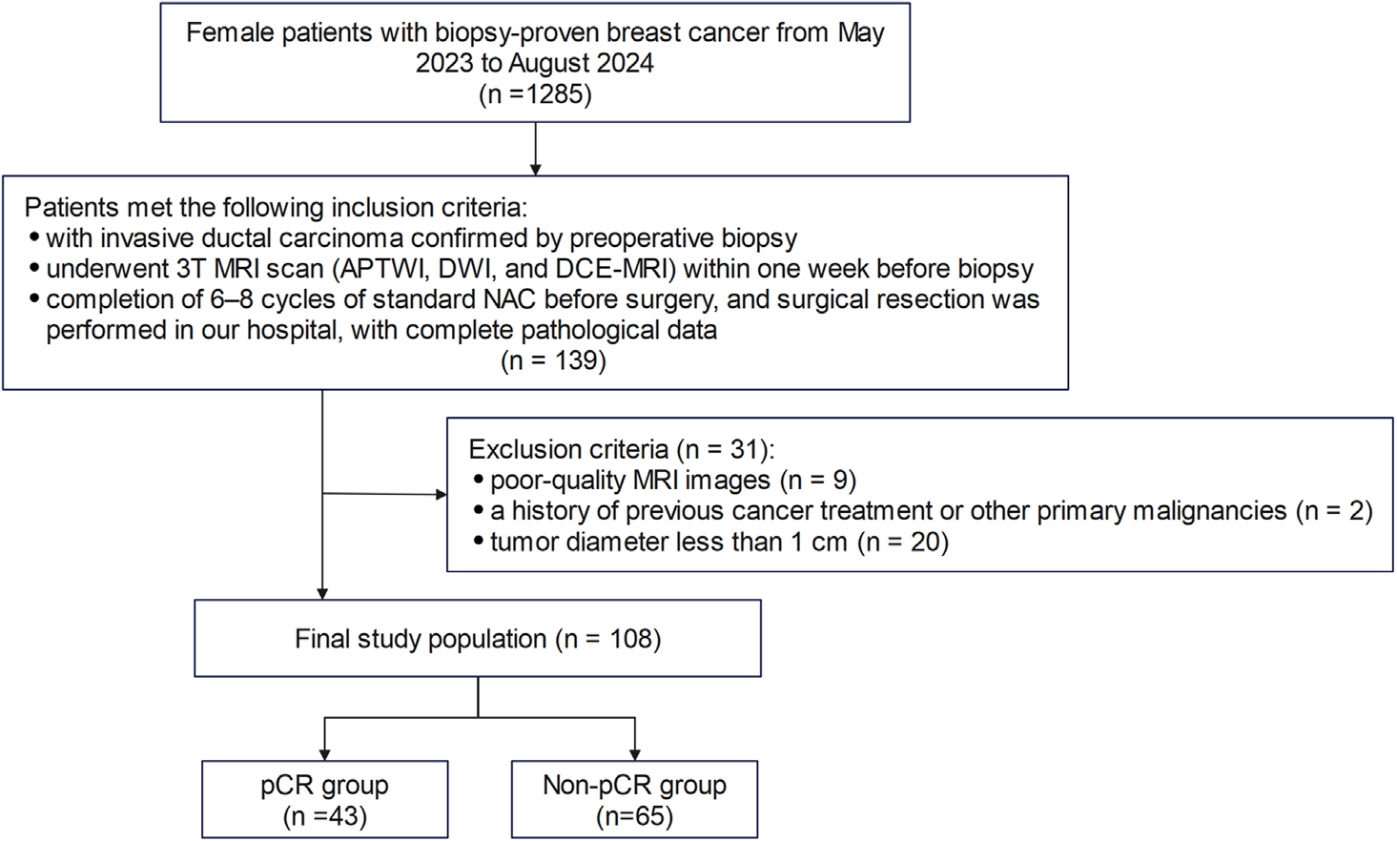
Flowchart of the patient selection process for this study cohort. APTWI, amide proton transfer‐weighted imaging; DWI, diffusion weighted imaging; DCE‐MRI, dynamic contrast‐enhanced MRI; NAC, neoadjuvant chemotherapy; pCR, pathologic complete response.

This retrospective study included consecutive female patients with biopsy‐confirmed breast cancer who received standard NAC between May 2023 and August 2024 at our hospital. Patients met the following inclusion criteria: (1) Invasive ductal carcinoma confirmed by preoperative biopsy; (2) Completion of a multiparametric 3 T MRI scan within 1 week before biopsy, including APTWI, DWI, and DCE‐MRI; and (3) Completion of 6–8 cycles of standard NAC before surgery, with surgical resection performed at our hospital and complete pathological data available. The exclusion criteria were as follows: (1) Poor‐quality MRI images; (2) History of previous cancer treatment or other primary malignancies; and (3) Tumor diameter of less than 1 cm. The study flowchart is presented in Figure 1.

### 2.2 Imaging Acquisition and Analysis

MRI was performed using a 3 T scanner (Ingenia CX, Philips Healthcare, Netherlands) with an 8‐channel phased‐array breast coil. For premenopausal patients, menstrual cycle effects were not considered due to treatment schedules. Imaging was conducted with patients in the prone position. The sequences included axial T1WI, fat‐suppressed T2WI, APTWI, DWI, and both axial and sagittal DCE‐MRI. The scanning parameters are detailed in Table S1. APTWI, acquired using two breath‐holds; DCE‐MRI, conducted after the rapid injection of gadoteric acid contrast agent (Gadoteracid, Gd‐DOTA, Jiangsu Hengrui Medicine, Jiangsu, China) at 1.5 mL/s (0.1 mmol/kg). A fat‐saturated gradient echo sequence was used to capture three‐dimensional (3D) T1‐weighted dynamic contrast‐enhanced images covering the entire breast, with 40 phases.

The APTWI was performed using a 3D fast spin‐echo sequence. During each acquisition, a dual radiofrequency (RF) saturation pulse with a B1 amplitude (rms) of 2 μT and a duration of 2 s was applied at specific frequency offsets. The water proton frequency was defined as 0 ppm, and RF saturation was applied at frequency offsets of ±2.7, ±3.5, and ±4.3 ppm to obtain the Z‐spectrum. A reference image was acquired with the saturation frequency set at −1540 ppm. Additionally, three different echo times were collected at the +3.5 ppm saturation frequency to generate B0 maps for voxel‐wise Z‐spectrum correction. The APT value was calculated by measuring the asymmetry of the magnetization transfer ratio (MTR) at ±3.5 ppm relative to the water resonance frequency, using the following equation: APTw% = MTRasym [Δ*ω* = +3.5 ppm] (%) = (*S* − Δ*ω* − *S*Δ*ω*)/*S₀* × 100%, where *S₀* is the water signal intensity at the −1540 ppm reference scan, and *S* − Δ*ω* and *S*Δ*ω* are the B0‐corrected water signal intensities at +3.5 ppm and −3.5 ppm, respectively.

### 2.3 Imaging Procedure and Analysis

Images were uploaded to the ISP workstation (Philips Healthcare) and independently analyzed by two radiologists with 9 and 20 years of MRI diagnostic experience, respectively. Using MR Permeability analysis software, APTWI, DWI, and DCE‐MRI data were processed to obtain APT, ADC, and silhouette images at 30 and 90 s post‐contrast injection. Two observers were blinded to the patients' lesion locations, pathology, and clinical results. The 3D regions of interest (ROIs) were delineated layer by layer over the solid components of the entire tumor using ITK‐SNAP software based on enhanced images. Areas showing cystic, necrotic, and hemorrhagic features were excluded. Histogram features of each quantitative parameter were extracted using custom Matlab code. The average values of each parameter derived from the two observers were calculated for subsequent statistical analysis.

### 2.4 Pathological Evaluation

Histopathological assessments, including hematoxylin–eosin staining and immunohistochemistry, were conducted using both post‐biopsy and postoperative pathology results. These evaluations were independently performed by two pathologists, one of whom was a senior pathologist with over 15 years of diagnostic experience. In cases of disagreement, consensus was reached through discussion.

Pre‐NAC biopsy specimens for all patients were analyzed according to the American Society of Clinical Oncology (ASCO)/College of American Pathologists (CAP) guidelines. Estrogen receptor (ER) and progesterone receptor (PR) positivity were defined as staining in ≥ 1% of tumor cells, while < 1% staining indicated negativity [22]. HER2 amplification was assessed using a scoring system: scores of 0 or 1+ indicated negativity, 2+ was equivocal, and 3+ was positive for overexpression. Equivocal cases (2+) were confirmed via fluorescence in situ hybridization (FISH) analysis [23]. Ki‐67 expression levels were categorized as high (≥ 30%) or low (< 30%) [24].

Based on the China Anti‐Cancer Association Committee of Breast Cancer Society (CACA‐CBCS) guidelines, invasive ductal carcinoma was classified into molecular subtypes: Luminal A, Luminal B (HER2‐positive), Luminal B (HER2‐negative), triple‐negative (TN), and HER2‐enriched according to ER, PR, HER2, and Ki‐67 expression levels. In this study, Luminal B (HER2‐positive) and Luminal B (HER2‐negative) were grouped together under Luminal B [25].

Following 6–8 cycles of NAC, all patients underwent surgical resection. Residual invasive tumor burden in the tumor bed post‐NAC was evaluated using the Miller Payne (MP) grading system. Grade 5 represented pCR, while grades 1–4 indicated non‐pCR.

### 2.5 NAC Treatment Regimen

All NAC regimens were administered according to the National Comprehensive Cancer Network (NCCN) Breast Cancer Guidelines and the Chinese Society of Clinical Oncology (CSCO) Breast Cancer Guidelines (2024 edition), which are widely recognized as the standard reference for breast cancer treatment. The specific NAC regimen for each patient was determined based on the pathological immunohistochemical profile and genetic expression of biopsy samples. The regimens of all patients included TCbHP (docetaxel + carboplatin + trastuzumab + pertuzumab), TCb (docetaxel + carboplatin), TCH (docetaxel + carboplatin + trastuzumab), PH (paclitaxel + trastuzumab) plus pyrotinib, and TE (docetaxel + epirubicin). Each cycle spanned 21 days, with a total of 6–8 cycles administered.

### 2.6 Statistical Analysis

Statistical analyses were performed using the R programming language (version 4.2). Inter‐observer consistency was evaluated using the intra‐class correlation coefficient (ICC), where ICC > 0.8 indicated good agreement. The Shapiro–Wilk test was used to assess whether continuous variables followed a normal distribution. Variables with normal distributions were presented as mean ± standard deviation, while non‐normally distributed variables were reported as median (interquartile range). Chi‐squared test or Fisher's exact test was used to compare the differences in clinical and pathological categorical variables, and independent samples *t*‐test or Mann–Whitney *U* test was used to compare the differences in the quantitative parameters obtained from histogram analysis of APT, ADC, and early enhanced silhouette images. Multivariate logistic regression using a stepwise method was conducted on parameters with significant differences to identify independent predictors of pCR after NAC. A predictive model for NAC efficacy was then developed. The diagnostic performance of each parameter and the predictive model was assessed using receiver operating characteristic (ROC) curves, and the area under the curve (AUC) values were compared using the DeLong test. A *p* value < 0.05 was considered statistically significant.

## 3 Results

### 3.1 Clinical, Pathological, and MRI Characteristics

A total of 108 patients were included in this study (mean age 49.05 ± 10.37 years; age range: 30–70 years). Among them, 43 patients were in the pCR group, and 65 were in the non‐pCR group. The clinical and MRI characteristics of all patients are presented in Table 1.

**TABLE 1:** Clinical, pathological, and MRI features of 108 patients.

	Total (*n* = 108)	pCR (*n* = 43)	Non‐pCR (*n* = 65)	*t*/*χ* ^2^	*p*
Age (years)	49.05 ± 10.37	49.95 ± 10.44	49.18 ± 9.85	0.388	0.699
Menopausal status					
Premenopausal	52 (48%)	20 (47%)	33 (51%)	0.077	0.782
Postmenopausal	56 (52%)	23 (53%)	32 (49%)		
Histological grade (HG)					
HG1‐2	76 (70%)	31 (72%)	45 (69%)	0.102	0.750
HG3	32 (30%)	12 (28%)	20 (31%)		
Lymph node metastasis					
Positive	66 (58%)	26 (60%)	40 (62%)	0.013	0.911
Negative	42 (39%)	17 (40%)	25 (38%)		
TNM stage					
T stage					
T1	11 (10%)	5 (12%)	6 (9%)	1.449	0.694
T2	77 (71%)	29 (67%)	48 (74%)		
T3	11 (10%)	6 (14%)	5 (8%)		
T4	9 (9%)	3 (7%)	6 (9%)		
N stage					
N0	45 (42%)	18 (42%)	27 (42%)	0.849	0.838
N1	42 (39%)	15 (35%)	27 (42%)		
N2	15 (14%)	7 (16%)	8 (12%)		
N3	6 (5%)	3 (7%)	3 (4%)		
M stage					
M0	108 (100%)	43 (100%)	65 (100%)	—	—
M1	0 (0%)	0 (0%)	0 (0%)		
Enhancement pattern					
Tumor‐like	93 (86%)	35 (81%)	58 (89%)	1.328	0.249
Non‐tumor‐like	15 (24%)	8 (19%)	7 (11%)		
Time‐signal curve					
Inflow	5 (5%)	2 (5%)	3 (5%)	0.363	0.834
Plateau	49 (45%)	21 (49%)	28 (43%)		
Outflow	54 (50%)	20 (46%)	34 (52%)		
Molecular subtype					
Luminal A	4 (4%)	1 (2%)	3 (5%)	—	**< 0.001**
Luminal B	51 (47%)	11 (26%)	40 (61%)		
HER2‐Enriched	23 (21%)	18 (42%)	5 (8%)		
TN	30 (28%)	13 (30%)	17 (26%)		
ER					
Positive	54 (50%)	11 (26%)	43 (66%)	17.040	**< 0.001**
Negative	54 (50%)	32 (74%)	22 (34%)		
PR					
Positive	44 (41%)	10 (23%)	34 (52%)	9.047	**0.003**
Negative	64 (59%)	33 (77%)	31 (48%)		
HER2					
Positive	40 (37%)	26 (60%)	14 (22%)	16.816	**< 0.001**
Negative	68 (63%)	17 (40%)	51 (78%)		
Ki‐67					
High expression	96 (89%)	42 (98%)	54 (83%)	4.203	**0.040**
Low expression	12 (11%)	1 (2%)	11 (17%)		

*Note:* The bold represents a statistically significant difference.

### 3.2 Inter‐Observer Consistency

The interobserver consistency for parameter measurements by the two radiologists was good. The ICC values for categorical clinical and MRI variables were all above 0.9. Similarly, the ICC values for quantitative parameters of MTRasym derived from histogram analysis of APT, ADC, and early enhanced silhouette images were all above 0.8. Consequently, the mean values measured by the two observers were used for subsequent statistical analysis.

### 3.3 Comparison of Histogram Features of Quantitative Parameters From APT, ADC, and Early Enhanced Silhouette Images in Breast Cancer Between pCR and Non‐pCR Groups

**FIGURE 2:**
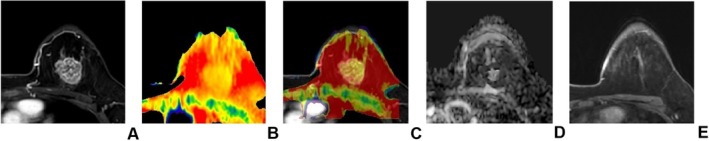
40‐year‐old woman with breast cancer achieved pCR after NAC. (A) Baseline T1WI enhanced imaging before NAC; (B) APT map; (C) T1WI enhanced and APT fusion diagram; (D) ADC map; (E) T1WI enhanced imaging after NAC. The mean ADC and MTRasym values measured by two radiologists were 0.61 × 10^−3^ mm^2^/s and 3.76%, respectively.

**FIGURE 3:**
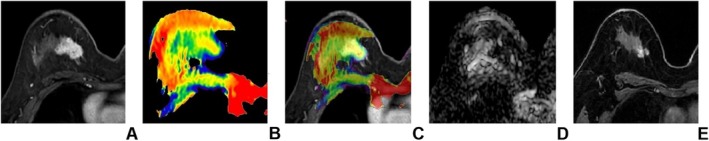
43‐year‐old woman with breast cancer achieved non‐pCR after NAC. (A) Baseline T1WI enhanced imaging before NAC; (B) APT map; (C) T1WI enhanced and APT fusion diagram; (D) ADC map; (E) T1WI enhanced imaging after NAC. The mean ADC and MTRasym values measured by two radiologists were 0.39 × 10^−3^ mm^2^/s and 2.15%, respectively.

In comparing histogram features of quantitative parameters obtained from pretreatment APT, ADC, and early enhanced silhouette images between pCR and non‐pCR groups, the mean, root‐mean‐square deviation (RMSE), and the 5th, 10th, 15th, 25th, 50th, 75th, 85th percentiles of MTRasym, along with 1st percentiles of ADC were significantly higher in the pCR group compared to the non‐pCR group (all *p* < 0.05) (Figures 2 and 3, Table 2). Conversely, the interquartile range of MTRasym and 10th percentiles of ADC were significantly lower in the pCR group (all *p* < 0.05) (Table 2). However, no statistically significant differences were observed in any histogram features of the 30‐s and 90‐s enhanced net increment or in other histogram features of MTRasym and ADC values between pCR and non‐pCR groups.

**TABLE 2:** Comparison of histogram features of quantitative MRI parameters of breast cancer between pCR and non‐PCR groups.

Histogram analysis features	pCR (*n* = 43)	Non‐pCR (*n* = 65)	*t*/*Z*	*p*
MTRasym value (%)				
5th percentile	0.83 ± 2.22	−0.54 ± 2.62	−2.81	0.006
10th percentile	1.29 ± 2.05	0.06 ± 2.23	−2.92	0.004
15th percentile	1.59 ± 1.94	0.44 ± 2.03	−2.93	0.004
25th percentile	2.00 ± 1.80	0.93 ± 1.89	−2.93	0.004
50th percentile	2.63 ± 1.63	1.74 ± 1.77	−2.65	0.009
75th percentile	3.20 ± 1.55	2.44 ± 1.78	−2.31	0.023
85th percentile	3.50 ± 1.56	2.81 ± 1.84	−2.01	0.047
Mean	2.55 ± 1.69	1.64 ± 1.82	−2.63	0.010
RMSE	3.02 ± 1.17	2.57 ± 1.15	−2.00	0.048
Interquartile range	1.04 (0.76, 1.51)	1.35 (0.96, 1.91)	−2.13	0.033
ADC value (×10^−3^ mm^2^/s)				
1st percentile	−0.15 (−0.45, 0.18)	−0.36 (−0.57, 0.08)	−2.040	0.041
10th percentile	0.13 (−0.23, 0.53)	0.26 (0.04, 0.78)	−1.996	0.046

*Note:* Indicators following a normal distribution were expressed as mean ± standard deviation, while those not following a normal distribution were expressed as median (interquartile range). Only quantitative parameters with statistically significant differences were displayed in this table.

Abbreviation: RMSE, root‐mean‐square deviation.

### 3.4 Diagnostic Performance of Histogram Features of MTRasym and ADC Values in Distinguishing Between pCR and Non‐pCR Groups

**FIGURE 4:**
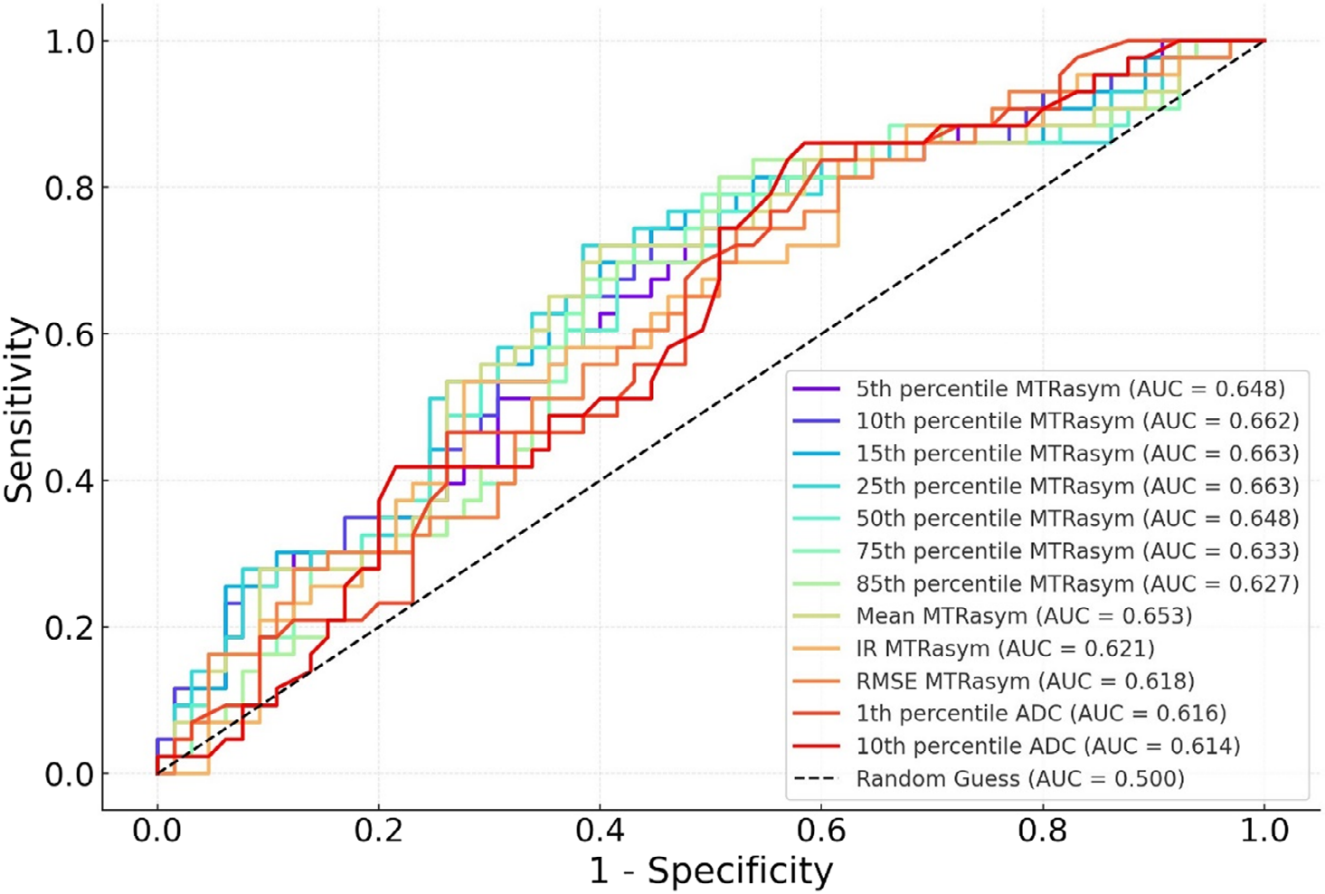
The ROC curves for distinguishing between the pCR and non‐pCR groups in breast cancer based on histogram features of MTRasym and ADC values.

The AUC values for predicting pCR after NAC in breast cancer based on pretreatment histogram analysis features of MTRasym, including mean, RMSE, interquartile range and the 5th, 10th, 15th, 25th, 50th, 75th, 85th percentile of MTRasym, along with 1st and 10th percentiles of ADC were shown in Table 3 and Figure 4.

**TABLE 3:** Diagnostic performance of quantitative MRI histogram features for predicting pCR in breast cancer.

Histogram analysis features	AUC	SE	95% CI	Cut‐off	Sensitivity (%)	Specificity (%)	Accuracy (%)
MTRasym value (%)							
5th percentile	0.648	0.0538	0.551–0.738	−0.45	79.07 (34/43)	49.23 (32/65)	61.11 (66/108)
10th percentile	0.662	0.0534	0.565–0.750	0.45	74.42 (32/43)	55.38 (36/65)	62.96 (68/108)
15th percentile	0.663	0.0538	0.565–0.751	0.64	74.42 (32/43)	55.38 (36/65)	62.96 (68/108)
25th percentile	0.663	0.0544	0.566–0.751	1.40	72.09 (31/43)	61.54 (40/65)	65.74 (71/108)
50th percentile	0.648	0.0547	0.550–0.737	2.09	72.09 (31/43)	56.92 (37/65)	62.96 (68/108)
75th percentile	0.633	0.0549	0.534–0.723	2.16	79.07 (34/43)	50.77 (33/65)	62.04 (67/108)
85th percentile	0.627	0.0549	0.529–0.718	2.55	81.40 (35/43)	49.23 (32/65)	62.04 (67/108)
Mean	0.653	0.0545	0.556–0.742	1.91	72.09 (31/43)	60.00 (39/65)	64.81 (70/108)
RMSE	0.618	0.0548	0.519–0.709	2.29	74.42 (32/43)	47.69 (31/65)	58.33 (63/108)
Interquartile range	0.621	0.0548	0.523–0.713	1.06	53.49 (23/43)	72.31 (47/65)	64.81 (70/108)
ADC value (×10^−3^ mm^2^/s)							
1st percentile	0.616	0.0542	0.518–0.708	−0.52	83.72 (36/43)	40.00 (26/65)	57.41 (62/108)
10th percentile	0.614	0.0546	0.515–0.706	−0.09	86.05 (37/43)	41.54 (27/65)	59.26 (64/108)

Abbreviations: 95% CI, 95% confidence interval; RMSE, root‐mean‐square deviation; SE, standard error.

### 3.5 Construction a Predictive Diagnostic Model for pCR After NAC in Breast Cancer

Univariate logistic regression analysis was conducted on parameters that showed statistically significant differences between the pCR and non‐pCR groups following NAC. These included the molecular subtype and immunohistochemical factors, such as ER, PR, HER2, and Ki‐67 expression, as well as histogram features of MTRasym and ADC values (Table 4). Factors with *p* < 0.05 were included in a multivariate logistic regression analysis with the forward stepwise regression method. The results indicated that ER‐negative, HER2‐positive status, and the 5th percentile MTRasym value were independent predictors of achieving pCR after NAC in breast cancer, with odds ratios (ORs) of 0.16, 7.25, and 1.35, respectively (Table 4).

**TABLE 4:** Univariate and multivariate logistic regression results.

Variables	Univariate logistic regression					Multivariate logistic regression				
	*β*	SE	*Z*	*p*	OR (95% CI)	*β*	SE	*Z*	*p*	OR (95% CI)
ER										
Negative					1.00 (Reference)					1.00 (Reference)
Positive	−1.74	0.44	−3.98	**< 0.001**	0.18 (0.07 ~ 0.41)	−1.85	0.51	13.28	**< 0.001**	0.16 (0.06 ~ 0.42)
PR										
Negative					1.00 (Reference)					
Positive	−1.29	0.44	−2.94	**0.003**	0.28 (0.12 ~ 0.65)					
HER2										
Negative					1.00 (Reference)					1.00 (Reference)
Positive	1.72	0.43	3.96	**< 0.001**	5.57 (2.38 ~ 13.04)	1.98	0.52	14.27	**< 0.001**	7.25 (2.59 ~ 20.25)
Ki‐67										
Low expression					1.00 (Reference)					
High expression	2.15	1.06	2.02	**0.044**	8.56 (1.06 ~ 68.87)					
MTRasym value (%)										
5th percentile	0.23	0.09	2.65	**0.008**	1.26 (1.06 ~ 1.50)	0.30	0.11	8.38	**0.004**	1.35 (1.10 ~ 1.66)
10th percentile	0.27	0.10	2.75	**0.006**	1.31 (1.08 ~ 1.59)					
15th percentile	0.29	0.11	2.75	**0.006**	1.34 (1.09 ~ 1.65)					
25th percentile	0.32	0.12	2.75	**0.006**	1.38 (1.10 ~ 1.73)					
50th percentile	0.31	0.12	2.51	**0.012**	1.37 (1.07 ~ 1.75)					
75th percentile	0.28	0.12	2.22	**0.026**	1.32 (1.03 ~ 1.69)					
85th percentile	0.23	0.12	1.95	0.051	1.26 (1.00 ~ 1.60)					
Mean	0.30	0.12	2.50	**0.013**	1.35 (1.07 ~ 1.71)					
RMSE	0.34	0.17	1.95	0.051	1.40 (1.00 ~ 1.97)					
Interquartile range	−0.69	0.33	−2.09	**0.037**	0.50 (0.26 ~ 0.96)					
ADC value (×10^−3^ mm^2^/s)										
1st percentile	0.55	0.36	1.50	0.134	1.73 (0.84 ~ 3.52)					
10th percentile	0.69	0.39	1.77	0.077	2.00 (0.93 ~ 4.29)					

*Note:* Bold represents a statistically significant difference.

Abbreviations: CI, confidence Interval; OR, odds ratio; RMSE, root‐mean‐square deviation.

**FIGURE 5:**
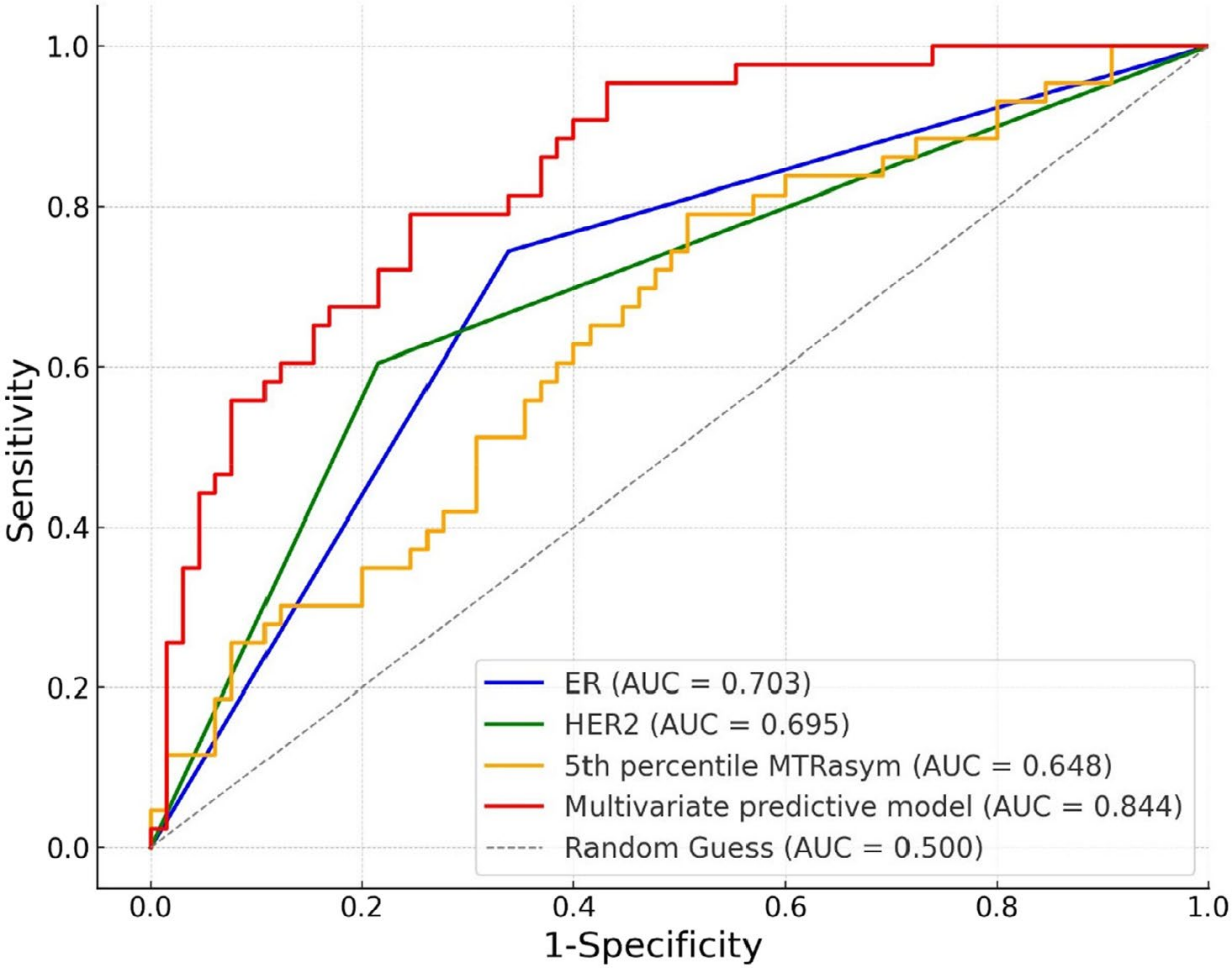
ROC curves of individual predictors and multivariate prediction model for predicting pCR after NAC in breast cancer.

Based on this multivariate logistic regression analysis, a predictive diagnostic model for pCR after NAC in breast cancer was constructed. The AUC for this combined multivariate predictive model was 0.844, with a sensitivity of 79.07%, specificity of 75.38%, and accuracy of 76.85%. Comparison of the diagnostic performance of ER‐negative, HER2‐positive, 5th percentile MTRasym value, and multivariate prediction model in predicting pCR after NAC in breast cancer showed that the diagnostic performance of the multivariate prediction model was significantly superior to that of each individual predictor (all *p* values < 0.001) (Table 5, Figure 5).

**TABLE 5:** Performance and comparison of independent predictors for predicting pCR after NAC in breast cancer.

Indicator	AUC (95% CI)	Sensitivity (%)	Specificity (%)	Accuracy (%)	Delong test^a^		
					ER‐ vs. Predictive model	HER2+ vs. Predictive model	5th percentile of MTRasym vs. Predictive model
ER	0.703 (0.607–0.787)	74.42 (32/43)	66.15 (43/65)	69.44 (75/108)			
HER2	0.695 (0.599–0.780)	60.47 (26/43)	78.46 (51/65)	71.30 (77/108)	*p* < 0.001	*p* < 0.001	*p* < 0.001
5th percentile MTRasym	0.648 (0.551–0.738)	79.07 (34/43)	49.23 (32/65)	61.11 (66/108)			
Multivariate predictive model	0.844 (0.761–0.906)	79.07 (34/43)	75.38 (49/65)	76.85 (83/108)			

^a^ The results of the Delong test are displayed only for indicators with statistically significant differences.

## 4 Discussion

This study investigated the value of histogram analysis applied to pre‐treatment APTWI, DWI, and early‐phase contrast‐enhanced silhouette images in predicting pathological response to NAC in breast cancer. The results demonstrated that the mean, RMSE, interquartile range, and the 5th, 10th, 15th, 25th, 50th, 75th, 85th percentile of MTRasym, along with 1st and 10th percentiles of ADC value could noninvasively predict the possibility of achieving pCR before treatment. This approach facilitates assessing the potential benefit of NAC, supports clinical decision‐making, and optimizes treatment strategies. Furthermore, combining APTWI features with preoperative biopsy‐based immunohistochemical markers, such as ER and HER2 expression, significantly improved diagnostic efficacy and accuracy in predicting pCR after NAC.

APTWI, a novel molecular imaging technique, remains in the exploratory stage for breast cancer applications. According to our literature review, this study represents the largest sample size to date using APTWI imaging to predict NAC efficacy in breast cancer. Our previous research demonstrated that MTRasym values from APTWI could differentiate four molecular subtypes of breast cancer, with certain trends: TN breast cancer exhibited the highest MTRasym values, followed by HER2‐enriched and Luminal B, while lower MTRasym values were more inclined to Luminal A (see Table S1). The cases in this study conformed to this trend. TN and HER2‐enriched breast cancers, categorized as ER‐negative cancers, are characterized by a large proportion of high‐grade tissues within lesions. Microscopically, these tumors display increased cellularity, few or absent tubular formations, syncytial clusters, large bare nuclei, and pronounced nuclear atypia [26, 27]. Such tumors demonstrate a high rate of cell division, rapid proliferation, and substantial angiogenesis, which includes a high concentration of hemoglobin and albumin, resulting in elevated protein diversity and levels. Consequently, these characteristics lead to an increase in APTWI signal.

In this study, the mean MTRasym value post‐NAC was significantly higher in the pCR group than in the non‐pCR group. First, this difference may be attributed to the 72% proportion of HER2‐enriched and TN subtypes in the pCR group compared to 34% in the non‐pCR group. Previous studies have indicated that HER2‐enriched and TN breast cancers typically have higher MTRasym values, which likely contributed to the higher mean MTRasym in this group. Conversely, Zhang [28] showed that pre‐NAC MTRasym values in the pCR group were lower than those in the non‐pCR group, differing from our findings. This discrepancy may be owing to differences in the distribution of molecular subtypes, particularly HER2‐enriched and TN cancers, which were not clarified by Zhang et al., thus limiting further analysis. Second, rapidly proliferating tumor cells are more susceptible to NAC‐induced damage than quiescent cells due to their reduced repair time. These tumors have high blood flow and permeability, increasing chemotherapy access and reducing hypoxia‐mediated drug resistance, thereby making them more responsive to NAC [29]. Chen et al. [30, 31] have also demonstrated that highly proliferative tumors exhibit higher tumor regression and pCR rates post‐NAC, which is consistent with our findings. Third, a large‐scale study by Raphael et al. [32, 33] found that breast cancers with low ER and PR expression status responded more effectively to NAC, with HER2‐enriched subtypes achieving the highest pCR rates. In contrast, Luminal A breast cancers, showed low sensitivity, with pCR rates as low as 0.3% [34]. In our study, only 25% Luminal A cases were pCR post‐NAC, and 72% of the pCR group consisted of HER2‐enriched and TN subtypes, consistent with previous research. Lastly, high‐grade tumors exhibit intense cellular metabolism; however, the chemical exchange between amide protons and free water, which is base‐catalyzed, typically decreases as pH decreases, theoretically lowering the MTRasym value [35]. However, Krikken et al. [36] indicated that at low pH, the measured number of tumor cells and protein concentration increased, indicating that protein and peptide concentrations are the primary factors influencing MTRasym values.

In this study, the interquartile range of MTRasym values in the pCR group was significantly lower than those in the non‐pCR group. This difference can be explained as follows: the interquartile interval reflects the dispersion degree of data and the existence of outliers; that is, a larger interquartile interval indicates a larger dispersion degree of data. In the non‐pCR group, a higher proportion of breast cancers exhibited cystic necrosis, resulting in a more heterogeneous distribution of protein and peptide concentrations within the tumor. Consequently, APT images of these tumors displayed greater dispersion in signal intensity. Although the ROI for the entire tumor in this study included only the solid components, small cystic or necrotic regions, which may have been difficult to detect visually, likely contributed to higher standard deviation and mean absolute deviation values. Furthermore, previous studies [37] have shown that in breast cancers with cystic necrosis, necrotic regions may limit drug distribution and penetration, making these areas less accessible to chemotherapy and generally reducing NAC sensitivity. Additionally, breast cancers with cystic necrosis are often high‐grade and may possess greater drug resistance at the cellular level, further contributing to the reduced efficacy of NAC.

DWI has been widely applied in tumor imaging. However, previous studies have shown varying results regarding its efficacy in predicting breast cancer response to NAC. In this study, no statistically significant difference was observed in the mean ADC values between the pCR and non‐pCR groups after NAC, consistent with findings by Nie et al. [38, 39]. Conversely, some researchers reported significantly lower ADC values in the pCR group compared to those in the non‐pCR group [40, 41], while others reported higher ADC values in the pCR group [40, 41]. These discrepancies may stem from several methodological differences. First, the approach to defining the ROI for breast tumor lesions varies significantly across studies, which can impact the results. For instance, Hottat et al. [39] demonstrated that using a 2D ROI in breast cancer lesions yielded an AUC of 0.821 for predicting pCR, whereas whole‐tumor 3D ROI measurements could not reliably predict pCR. To ensure consistency with the full‐tumor measurement range used in APTWI and to capture the overall tumor characteristics, this study employed a whole‐tumor 3D ROI approach. Second, differing opinions exist regarding whether to include cystic or necrotic areas in the ROI. Since the solid components of the tumor more accurately reflect the biological properties of tumor cells, this study focused on delineating only the solid portions, using enhancement regions on DCE‐MRI as a guide to avoid including cystic or necrotic areas as much as possible. Lastly, variations in the choice of b‐values in DWI protocols across studies could also lead to inconsistencies. Although this study found higher 1st percentile and lower 10th percentile ADC values in the pCR group than in the non‐pCR group, the diagnostic performance remained suboptimal, with the specificity of only 40.00% and 41.54%. Therefore, it appears that pre‐treatment ADC value is less predictive of pCR in patients with breast cancer undergoing NAC than APTWI.

This study also analyzed independent predictors of pCR following NAC in breast cancer. A combined diagnostic prediction model incorporating the 5th percentile MTRasym value with ER and HER2 expression demonstrated strong predictive performance (AUC = 0.844), providing a relatively accurate prediction of the likelihood of achieving pCR after NAC. Notably, the model outperformed any single parameter, underscoring the added value of combining imaging biomarkers with immunohistochemical profiles. These findings supported the potential clinical utility of this model as an effective, non‐invasive tool to guide and optimize individualized treatment strategies. It should be noted that our predictive model was designed to predict composite pCR (ypT0/Tis ypN0), which reflected treatment response in both the breast and axillary lymph nodes. While clinically standard, this definition did not differentiate responses between the two sites. Indeed, in our cohort, among the 65 patients in the non‐pCR group, 13 patients (21.5%) achieved axillary pCR despite having residual disease in the breast. The ability to specifically predict lymph node response could significantly influence axillary surgical management by avoiding axillary lymph node dissection in patients without metastatic involvement confirmed by sentinel lymph node biopsy, thereby markedly reducing the risk of complications such as upper limb lymphedema. Future large‐scale prospective studies are warranted to develop dedicated models for axillary response prediction based on baseline APTWI and identified clinical predictors.

### 4.1 Limitations and Future Scope

This study had several limitations. First, the relatively small, single‐center cohort may limit the generalizability of our findings, requiring future validation in larger multicenter cohorts. Second, the inherent spatial resolution and signal‐to‐noise ratio limitations of APTWI and DWI precluded the clear visualization of smaller lesions, necessitating the inclusion of only those breast cancers exceeding 1 cm in size to safeguard data accuracy. Third, the limited sample size prevented analysis of the impact of different NAC regimens on outcomes; however, all patients received standard NAC protocols according to guidelines without additional targeted or immunotherapies, thereby minimizing potential confounding from treatment heterogeneity. Fourth, our study was limited to pre‐treatment parameters, whereas longitudinal assessment during NAC could provide additional insights into the dynamic evolution of treatment response.

Future research should focus on multi‐parametric and multi‐center validation of APTWI‐based predictive models, integration with radiomics or deep learning approaches, and exploration of temporal changes in imaging biomarkers during NAC. Such efforts could further enhance the accuracy and clinical utility of non‐invasive imaging tools in guiding personalized treatment for breast cancer patients.

## 5 Conclusion

Pre‐treatment histogram analysis of APTWI shows potential diagnostic value in distinguishing between pCR and non‐pCR outcomes in breast cancer after NAC. This approach offers a non‐invasive, pre‐treatment estimation of the likelihood of therapeutic benefit for patients with breast cancer. A prediction model based on pre‐treatment APTWI histogram analysis combined with ER and HER2 expression statuses greatly improves diagnostic accuracy for predicting pCR after NAC. Additionally, this model could serve as an early indicator to identify patients unlikely to benefit from NAC, enabling timely discontinuation of ineffective treatments and the initiation of alternative therapies. Ultimately, this approach helps clinicians develop personalized treatment plans, potentially improving survival rates.

## Author Contributions

M.X. collected, analyzed and count the data, and was a major contributor in writing the manuscript; D.S. scanned patient images and collected data; C.X., J.L., R.Z. and X.C. provided guidance on study design and article writing; Z.S. provided technical guidance on APTWI and histogram analysis; Y.W. provided technical support for MRI; X.C. also provided technical and team support; J.Q. supervised the study, contributed to the study design, data interpretation, and manuscript revision.

## Funding

This work was supported by the Joint Construction Project of the Medical Science and Technology Research Program of Henan Province, China (LHGJ20240124) and the National High Level Hospital Clinical Research Funding and National Cancer Center Climbing Fund (No. NCC202416005).

## Conflicts of Interest

The authors declare no conflicts of interest.

## Supporting information


**Table S1:** Scanning parameters.

## Data Availability

The data that support the findings of this study are available on request from the corresponding author. The data are not publicly available due to privacy or ethical restrictions.
