# Supplementary material for: Pretreatment Amide Proton Transfer‐Weighted Imaging Histogram Analysis Combined With ER‐Negative and HER2‐Positive Expression Predicts Pathologic Complete Response After Neoadjuvant Chemotherapy in Breast Cancer
Source: Cancer Med. 2025 Dec 7;14(23):e71420. doi: 10.1002/cam4.71420 (PMC12682392; doi:10.1002/cam4.71420)
Supplement: Supplementary file 1 — Table S1: Scanning parameters. [file CAM4-14-e71420-s001.docx]

**Supplementary table**

**Table S1** Scanning parameters

| Parameters | T1WI | Fs-T2WI | APT | DWI | DCE |
| --- | --- | --- | --- | --- | --- |
| Postion | Axial | Axial | Axial | Axial | Axial |
| TR/TE (ms) | 5.5/2.3 | 4000/70 | 6350/8.3 | 6000/52 | 3.2/1.32 |
| Slice thickness (mm) | 1 | 4 | 6 | 4 | 1.5 |
| Slice gap (mm) | 0 | 0.8 | 0 | 0.8 | 0 |
| Field of view (mm²) | 260×340 | 260×340 | 260×140 | 200×340 | 340×340 |
| Matrix | 260×340 | 260×340 | 168×130 | 78×136 | 227×227 |
| B value (s/mm²) | / | / | / | 0,800 | / |
| Flip angle (°) | 120 | 120 | 120 | NA | 15 |
| Scan time (min:s) | 1:08 | 2:27 | 4:11 | 3:15 | 6:49 |
